# Selenium nanoparticles as a biological safe endodontic irrigant improving root canal sealing

**DOI:** 10.1186/s12903-025-07535-5

**Published:** 2026-01-09

**Authors:** Salma MH. Genena, Aya S Sedik, Marwa M Essawy, Nancy M. El Halfawy, Mahmoud R. AboElSeoud

**Affiliations:** 1 Department of Conservative, Faculty of Dentistry, Alexandria University, Alexandria, 21512 Egypt; 2 Department of Oral Biology, Faculty of Dentistry, Alexandria University, Champollion Street, Azarita, Alexandria, 21512 Egypt; 3 Department of Oral Pathology, Faculty of Dentistry, Alexandria University, Alexandria, 21512 Egypt; 4 Center of Excellence for Research in Regenerative Medicine and Applications (CERRMA), Faculty of Medicine, Alexandria University, Alexandria, 21512 Egypt; 5 Department of Botany and Microbiology, Faculty of Science, Alexandria University, Alexandria, 21512 Egypt

**Keywords:** Biofilm elimination, Cytocompatibility, *Enterococcus faecalis*, Root canal disinfection, Sodium hypochlorite

## Abstract

**Objectives:**

Elimination of *Enterococcus faecalis* (*E. faecalis*) biofilms from root canals remains a critical challenge in root canal treatment. Sodium hypochlorite (NaOCl) is the gold standard due to its potent antimicrobial and tissue-dissolving properties. However, its cytotoxic effects and risk of dentinal erosion necessitate the development of safer alternatives. This study evaluates the antibacterial efficacy, cytocompatibility, and dentinal interactions of selenium nanoparticles (SeNPs) as a potential endodontic irrigant.

**Methods:**

SeNPs were synthesized using an ascorbic acid-mediated approach and characterized for physicochemical properties. Antibacterial activity was assessed against *E. faecalis* (ATCC 47077) using the minimum inhibitory concentration (MIC) method. Cytotoxicity was evaluated on oral epithelial cells (OECs) and human gingival fibroblasts (HGFs) using an MTT assay. Extracted single-rooted teeth were inoculated with *E. faecalis* biofilms for 21 days and divided into NaOCl and SeNPs groups. After irrigation, dentinal surfaces were examined using scanning electron microscopy (SEM). Bacterial scores were analyzed using Kruskal-Wallis test, followed by Dunn–Bonferroni post hoc analysis, setting *p* < 0.05 to be significant.

**Results:**

SeNPs exhibited spherical morphology with an average size of 93.15 nm and good colloidal stability. MIC determination revealed effective antibacterial activity at ~ 128 µg/mL. SeNPs demonstrated an acceptable safety margin relative to the antibacterial concentration with an IC50 of 626 µg/mL on OECs and 809 µg/mL on HGFs, supporting their potential for clinical use. SEM revealed a relative bacterial reduction between irrigants, NaOCl (2.5%) and SeNP (128 µg/mL), where NaOCl-treated dentin disclosed complete removal of the smear layer with open tubules. Meanwhile, SeNP-treated dentin exhibited partial tubule occlusion and scattered nanoparticle deposits.

**Conclusion:**

SeNPs demonstrate promising antimicrobial efficacy, biocompatibility, and favorable dentinal interactions. Their ability to reduce bacterial load while potentially sealing dentinal tubules highlights their suitability as a safer, multifunctional alternative to conventional irrigants for root canal disinfection.

## Introduction

Successful root canal treatment depends on the effective elimination of microbial biofilms from the root canal system, with *Enterococcus faecalis* (*E. faecalis*) being one of the key pathogens associated with persistent periapical infections and treatment failure. Despite its sparsity in primary infections, *E. faecalis* is strongly associated with persistent and refractory endodontic cases due to its ability to penetrate dentinal tubules, survive harsh canal conditions, and form resistant biofilms. These virulence traits make it a clinically relevant benchmark organism for evaluating novel irrigants [1, 2].

Conventional irrigants, such as sodium hypochlorite (NaOCl), are widely used for root canal disinfection due to their broad-spectrum antimicrobial activity and tissue-dissolving properties [3]. However, it is associated with several drawbacks, including cytotoxicity, unpleasant taste, and the potential to weaken dentinal structures [4, 5]. These limitations have prompted the search for safer, more biocompatible alternatives with equivalent or superior antimicrobial efficacy.

Recent advances in nanotechnology have expanded the use of nanoparticles as promising agents for various biomedical applications, particularly in antimicrobial therapy [6, 7]. Despite this progress, the integration of nanomaterials as root canal irrigants remains relatively underexplored. Barriers such as the cost of large-scale synthesis, challenges in standardization, and characterization methods may limit their routine application in endodontics [8]. Nevertheless, nanoparticles possess unique physicochemical properties that differ significantly from their bulk counterparts, including enhanced surface reactivity and antimicrobial potential [9]. These attributes warrant further investigation into their safety profile and antibacterial efficacy, with the long-term goal of identifying nanomaterials that could serve as safer and more effective alternatives to conventional irrigants such as sodium hypochlorite (NaOCl).

Identifying a nanomaterial that combines antimicrobial potential with safety and cost-effectiveness is crucial for developing a viable alternative to NaOCl as a root canal irrigant. Among the candidates investigated, selenium nanoparticles (SeNPs) have attracted considerable attention due to their potent antimicrobial activity, antioxidant capacity, and relatively low toxicity [10]. Selenium, an essential trace element, serves as the active site of selenocysteine-containing proteins (selenoproteins), a group of enzymes that act as pivotal regulators of redox homeostasis in the body [11, 12]. When reduced to the nanoscale, selenium exhibits enhanced bioavailability, broadening its therapeutic window by increasing the release of reactive oxygen species (ROS), leading to cell membrane damage, inhibition of amino acid synthesis, and the blockage of DNA replication [13, 14].

The global market forecast of SeNPs is experiencing robust growth driven by increasing demand across diverse applications. Focusing on their antimicrobial properties, SeNPs synthesized via different techniques, including both chemical and eco-friendly approaches, have demonstrated efficacy against a broad spectrum of microorganisms, including resistant bacterial strains [15–18], Within the diversity of oral microbiota, SeNPs have exhibited antimicrobial activity against *Candida albicans*, *Streptococcus mutans*, *Porphyromonas gingivalis*, and *E. faecalis*, in both planktonic and biofilm states [19–21]. Considering the clinical need for root canal irrigants that balance antimicrobial efficacy with reduced cytotoxicity, SeNPs represent a promising candidate for further investigation, particularly targeting *E. faecalis*, the persistent pathogen in endodontic infections.

The current study aims to synthesize and characterize SeNPs to evaluate their antimicrobial efficacy as a novel endodontic irrigant against *E. faecalis* in comparison with the gold standard NaOCl. To test our hypothesis, the SeNP cytocompatibility profile on oral epithelial and gingival fibroblasts lineages revealed a biologically safe range with an earlier antibacterial efficacy. Additionally, scanning electron microscopy (SEM) results provided insightful evidence to reject the null hypothesis that SeNPs would exhibit no significant difference in antimicrobial efficacy or dentinal surface effects compared to NaOCl when used as an endodontic irrigant.

## Materials and methods

### Sample size estimation

The minimal sample size was calculated using GPower version 3.1.9.2 [22] based on a previous study aimed at determining the efficacy of 4 irrigation systems in eliminating bacteria in root canals, particularly in dentinal tubules. Sample size estimation was based on detecting the difference in bacterial reduction percentage [23]. Adopting a power of 80% (*β* = 0.20) to detect a standardized effect size in bacterial reduction percentage (primary outcome), and a level of significance of 5% (α error accepted = 0.05), the minimum required sample size was 8 teeth *per* group [24].

### Preparation of teeth and inoculation with E. faecalis

Twenty-four extracted human single-rooted teeth with mature apices and straight canals were selected for this study. Thorough cleaning of teeth from soft tissue residues was performed and stored in 0.1% thymol solution until use to prevent microbial contamination [25]. Standardized access cavities were prepared using a high-speed round bur and a safe-ended diamond fissure bur under constant water cooling to minimize heat generation and prevent structural damage [26].

A standardized bacterial suspension of *E. faecalis* (ATCC 47077; Rockville, MD, USA) was prepared by culturing the strain in brain heart infusion (BHI) broth (HiMedia, India) for 24 h at 37 °C, followed by adjustment to a concentration of 1 × 10⁸ CFU/mL using a spectrophotometer (Fisher Scientific, USA, OD₆₀₀) [27]. The root canals were filled to the orifice level with freshly prepared *E. faecalis* suspension using sterile 1 mL insulin syringes fitted with 30 G needles to ensure precise inoculation [28].

Following inoculation, teeth were placed individually in a sterile 15 mL polypropylene tube containing 10 mL of sterile BHI broth and incubated statically at 37 °C in 100% humidity for 21-days to promote bacterial colonization on the canal walls and penetration into the dentinal tubules, as described in previous biofilm formation models [29]. To maintain bacterial viability and simulate clinical biofilm development, 5 mL aliquots of the culture medium were replaced with fresh sterile BHI broth every 3-days.

Root canal instrumentation was performed using a rotary nickel-titanium system to a standardized apical preparation size of 25 with a 4% taper, ensuring uniform canal preparation across all specimens. The working length was established by reducing the decoronated tooth length by 1 mm. A #15 K-file (Mani, Tochigi, Japan) was gently introduced to the estimated length, and radiographic verification was performed. The remaining roots were then mechanically prepared using the ProTaper Next rotary system (Dentsply Sirona Maillefer, Ballaigues, Switzerland) up to size X3 (0.30/7%) [30].

### Preparation and characterization of seNPs

Sodium selenite anhydrous (Na_2_SeO_3_, #90214-G25) was purchased from SDFCL-SD Fine-Chem Limited, India, while L-ascorbic acid (#50-81-7) was available from Alpha-Chemika, India. Polyvinyl pyrrolidone (PVP, Mw 40,000, #9003-39-8) was available from Sigma, USA.

The preparation of SeNPs followed a chemical reduction technique, where 10 mg Na_2_SeO_3_ was dissolved in 30 mL deionized water (DIH_2_O) containing 1% (w/v) PVP. Then, 3.5 mL of DIH_2_O, dissolving 35 mg L-ascorbic acid, was added dropwise. Changing the color to reddish-orange designates the formation of selenium nanosuspension after stirring for 30 min. Purification was done through centrifugation at 15,000 rpm for 15 min, followed by decanting the supernatant and air-drying [31].

Preliminary SeNPs characterization was through UV-Vis spectrophotometer (Nanodrop, DeNovix, DS-11 FX+, US), with a dilution ratio of 1:1. Meanwhile, dynamic light scattering (DLS) depiction to analyze particle size and zeta potential of SeNPs were determined using Zetasizer Nano ZS (Malvern Instruments, Worcestershire, UK) at a dilution ratio of 1:4. Transmission electron microscope (TEM; JOEL, JSM-6360LA, Japan) was carried to analyze the morphology and size of SeNPs [32].

### Appraisal of cytotoxicity

The cytotoxicity of NaOCl and SeNPs was evaluated on human oral epithelial primary cell culture (OEC, 36063-01, Celprogen Inc., USA) and human gingival fibroblasts (HGFs, isolated [33] and donated by the Center of Excellence for Research in Regenerative Medicine and Application; CERRMA). Cultivation of cell lines was in Dulbecco Modified Eagle Medium (DMEM high glucose, #DMEM-HPSTA) supplemented with 10% fetal bovine serum (#FBS-16 A) and 1% antibiotics (penicillin/streptomycin, #PS-B), all agents were from Capricorn Scientific GmbH (Germany). With a 9000-seeding density, cells were treated for 24 h with serial concentrations of NaOCl (0.01 to 1% v/v) and SeNPs (1 to 5 mg/mL). Then, each well received 100 µL MTT (3-(4,5-dimethythiazol-2-yl)-2,5-diphenyltetrazolium bromide, #20395.01, SERVA Electrophoresis GmbH, Germany). After 3–4 h incubation, 100 µL/well dimethyl sulfoxide (#67-68-5, Thermofisher Scientific, USA) was added in darkness to dissolve formazan crystals, where their optical density was quantified by an ELISA reader (Infinite F15 TECAN, Switzerland) at 570 nm, normalizing the cell response to untreated control cells [34].

### Agar well diffusion assay

The agar well diffusion method was employed to assess the in vitro antibacterial activity of SeNPs against *E. faecalis*. A standardized bacterial suspension (1 × 10^6^ CFU/mL) was prepared from an 18 h culture of *E. faecalis* grown in Mueller-Hinton broth (MHB; Oxoid, Hampshire, UK) and uniformly inoculated onto Mueller-Hinton agar plates using a sterile swab. Wells of 6 mm diameter were aseptically punched into the agar and filled with 100 µL of sterile SeNP solution.

Ampicillin (100 µL) served as a positive control, and sterile distilled water (qH₂O) was used as a negative control. The plates were incubated aerobically at 37 °C for 24 h. Following incubation, the diameters of the zones of inhibition were measured in millimeters using a digital caliper. The inhibition zones were compared between groups to determine the antibacterial efficacy [35].

### Assessing MIC of SeNPs

The MIC of SeNPs against *E. faecalis* was determined in vitro using the standard broth microdilution method following guidelines of the Clinical and Laboratory Standards Institute (CLSI). Briefly, 2-fold serial dilutions of sterile well dissolved SeNPs were prepared in sterile 96-well polystyrene microtiter plates (Thermo Scientific, UK) using Mueller–Hinton broth (MHB; Oxoid, Hampshire, UK), with final concentrations ranging from 1 to 1,024 µg/mL.

A standardized bacterial suspension of *E. faecalis* (ATCC 47077) was prepared by adjusting the optical density to 0.08–0.10 at 625 nm, corresponding to approximately 1 × 10^6^ CFU/mL. An aliquot of 50 µL of the bacterial inoculum was added to each well containing 50 µL of the SeNPs dilution, resulting in a final volume of 100 µL *per* well.

For validation of the experimental conditions, the negative control wells contained only broth and SeNPs (without bacteria). Meanwhile, positive control wells had bacteria without SeNPs. The plates were incubated aerobically at 37 °C for 24 h. Following incubation, bacterial growth was assessed by visual inspection and confirmed by measuring optical density at 600 nm using a microplate reader. The MIC was defined as the lowest concentration of SeNPs at which no visible bacterial growth was observed, consistent with CLSI guidelines. The antimicrobial selectivity index of SeNPs was calculated by dividing the cellular IC50 over antibacterial MIC [36].

### Irrigation protocol

Following the incubation period and standardized root canal preparation, the specimens were divided randomly into 3 experimental groups based on the irrigant used. Group I: Negative control group, where teeth were left unirrigated. Group II: Positive control group, where 2.5% NaOCl (DentaPro, New Jersey, USA) was applied in a total volume of 20 mL *per* specimen, consistent with volumes used in previous root canal disinfection studies [37]. Group III: The experimental group, where SeNP suspension of 128 µg/mL was applied in a total volume of 20 mL *per* specimen. The concentration of SeNPs was determined based on the MIC assay results, ensuring effective antimicrobial activity against *E. faecalis*.

Irrigation was performed using sterile 5 mL syringes equipped with 30 G side-vented irrigation needles (NaviTip, Ultradent, South Jordan, Utah, USA). The irrigant was delivered incrementally in 5 mL portions, with the needle positioned 1 mm short of the working length to facilitate optimal irrigant penetration and minimize apical extrusion. A gentle in-and-out motion was applied during irrigation to enhance the distribution of the irrigant within the canal [38]. Following irrigation, we rinsed the canals with 5 mL of sterile distilled water to remove residual irrigants and then dried them with sterile paper points before SEM evaluation.

### SEM analysis

To evaluate the effect of the irrigation protocols on bacterial elimination and dentinal surface morphology, after completion of the irrigation protocols, the specimens were immediately fixed in 4% buffered formalin to preserve dentinal and microbial structures and prevent any post-treatment alterations. This fixation protocol has been widely recommended for SEM preparation to ensure optimal preservation of surface morphology. Afterwards, the specimens were longitudinally split along the buccolingual axis using a diamond disc under water cooling to expose the internal root canal surface. The total canal length was measured with a digital caliper and divided into three standardized thirds: coronal, middle, and apical. For this study, SEM evaluation was focused on the middle and apical thirds, as they represent the most clinically relevant and challenging regions for irrigant penetration. The samples were dehydrated in ascending grades of ethanol (50%, 70%, 90%, and 100%), sputter-coated with gold, and examined under a SEM (Joel SEM, JSM-IT200) [39].

Representative photomicrographs were captured from the middle and apical thirds of each canal at a magnification of ×4000. At least two fields per region were imaged for each specimen. This standardized mapping ensured reproducibility of image acquisition and consistent comparative evaluation of dentinal surface morphology and bacterial presence across the different experimental groups. The presence of residual *E. faecalis* biofilm, bacterial adherence within dentinal tubules, and alterations in dentinal surface morphology were all assessed qualitatively. Quantitative evaluation of bacterial presence was performed using a standardized scoring system, where score 0 = no bacteria; score 1 = few scattered bacteria; score 2 = moderate bacterial presence; score 3 = many bacterial cells; and score 4 = dense bacterial colonization [7, 10]. To ensure inter-examiner reliability, all analyses were conducted by 2 blinded examiners.

### Statistical analysis

To ensure reproducibility, the MIC assay was conducted in duplicate across 2 independent experiments. Cytotoxicity testing was performed in triplicate for each of three biological replicates. The dose-response curves of NaOCl and SeNPs treating OEC and HGFs were analyzed using GraphPad Prism (Version 8.0, GraphPad Software, San Diego, CA, USA) *via* nonlinear regression analysis to calculate the half-maximal inhibitory dose (IC50).

Shapiro-Wilk test, followed by the Levene test, verified the homoscedasticity of the bacterial inhibition zone, which was analyzed using a one-way ANOVA test, followed by the Tukey multiple comparisons test. Meanwhile, the heteroscedastic SEM bacterial scores were analyzed using a non-parametric Kruskal-Wallis test, followed by Dunn–Bonferroni post hoc analysis for pairwise comparisons. Data were expressed as median score. A *p* < 0.05 was considered statistically significant. All analyses were conducted using IBM SPSS Statistics software (version 26.0; IBM Corp., Armonk, NY, USA) [40]. For inter-examiner reliability, the SEM-based bacterial scores obtained by 2 independent examiners were assessed by RStudio (4.4.2) using Cohen’s kappa statistics. Interpretation of agreement strength followed the Landis and Koch scale, which categorizes kappa values as slight (0.01–0.20), fair (0.21–0.40), moderate (0.41–0.60), substantial (0.61–0.80), and almost perfect (0.81–1.00) [41].

## Results

### Dose-dependent cytotoxicity and antimicrobial profile of SeNPs

**Fig. 1:**
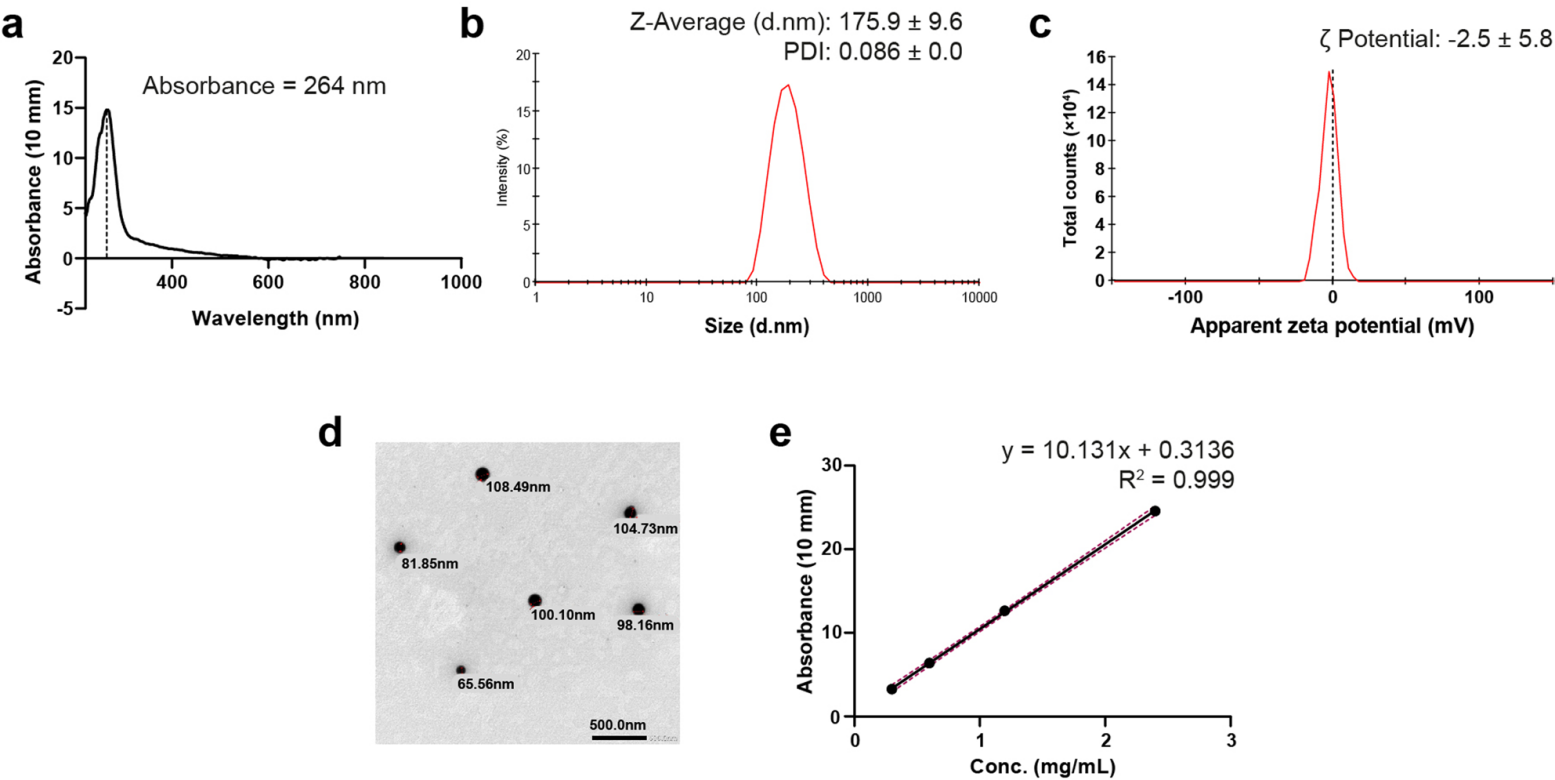
Optical-physical characterizations of PVP-SeNPs. **a** UV-Vis spectrophotometer, (**b**) and (**c**) Dynamic light scattering, (**d**) TEM, and (**e**) standard curve depict the synthesis of unimodal nanoparticles with an even narrow size range

The UV-Vis spectrum of the chemically reduced PVP-stabilized SeNPs revealed the synthesis of homogenously monomodal distributed nanoparticles with a smooth, regular, narrow peak at 262 nm (Fig. 1a). Characterizing the size and surface charge of PVP-SeNPs, DLS displayed a uniform peak with an average size of 175.9 ± 60.32 nm and a potential of -2.5 ± 5.88 mV (Fig. 1b, c). The low polydispersity index of 0.086 ± 0.01 was translated in the TEM analysis as widely dispersed nanospheres with an actual size reduced to 93.15 ± 16.3 nm (Fig. 1d). Meanwhile, the standard curve in Fig. 1e demonstrates almost a precise linear relation between PVP-SeNP concentration and the consistent UV-Vis absorbance.

**Fig. 2:**
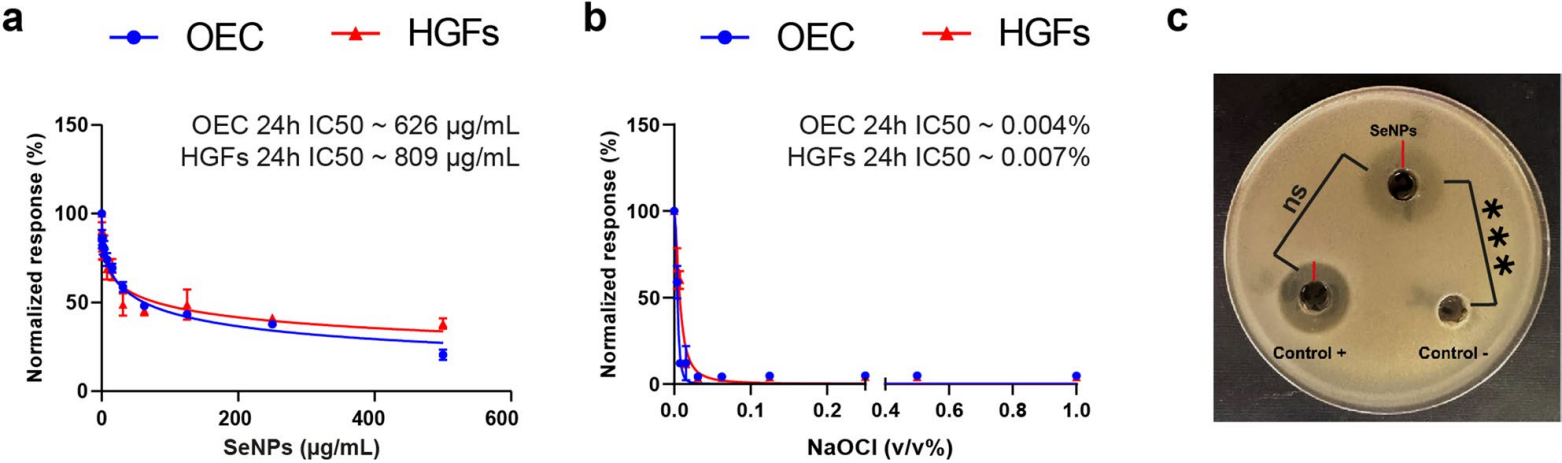
Cytological and antibacterial activity of SeNPs. The dose-dependent curves of (**a**) SeNPs and (**b**) NaOCl on OEC and HGFs reveal the safer cytological range of SeNPs over the acute toxicity of NaOCl on both cell lineages, with slight resistance of fibroblasts over epithelial cells. (**c**) The agar diffusion assay shows the inhibition zones (red lines) after 24 h of E. faecalis incubation with SeNPs, taking ampicillin as a positive control and sterile qH2O as a negative control. One-way ANOVA followed by Tukey post hoc test reveals a significant difference in bacterial inhibition zones between SeNPs and the negative control (***p < 0.001), in contrast to the non-significant difference (ns, *p* > 0.05) observed between SeNPs and the positive antibacterial control

Cytologically, PVP-SeNPs exhibited a dose-dependent trend with a consistent reduction in cell response upon increasing nanoparticle concentration in both cell lineages, OEC and HGFs. Moreover, the HGFs were more resistant to SeNPs, recording a slightly higher IC50 of ~ 809 µg/mL than the OEC IC50 of ~ 626 µg/mL (Fig. 2a). Testing the cytological response to NaOCl, both epithelial and fibroblasts were extremely sensitive to NaOCl even at very low volume concentration with an early drop of viability at 0.004% for OEC and 0.007% for HGFs (Fig. 2b).

Microbiologically, MIC of SeNPs at 128 µg/mL effectively inhibited *E. faecalis* growth after 24 h of incubation. The early microbial susceptibility of *E. faecalis* to low PVP-SeNPs dose compared with cellular IC50. The antibacterial selectivity index of PVP-SeNPs ranged from ~ 4.7 when compared with OEC IC50 to a slightly increased index of ~ 6.3 in relation to HGFs IC50. The MIC values were reproducible across 2 independent experiments. Additionally, the SeNPs solution demonstrated antibacterial activity in the agar well diffusion assay against *E. faecalis*, with a distinct zone of inhibition measuring 18.3 ± 1.5 mm in diameter, comparable to 16.3 ± 0.5 mm the inhibition capacity of the positive control (*p >* 0.05, Fig. 2c). This result reinforces the strong antimicrobial potential of the synthesized SeNPs and supports their application as a novel endodontic irrigant.

### Bacterial endodontic inoculation

**Fig. 3:**
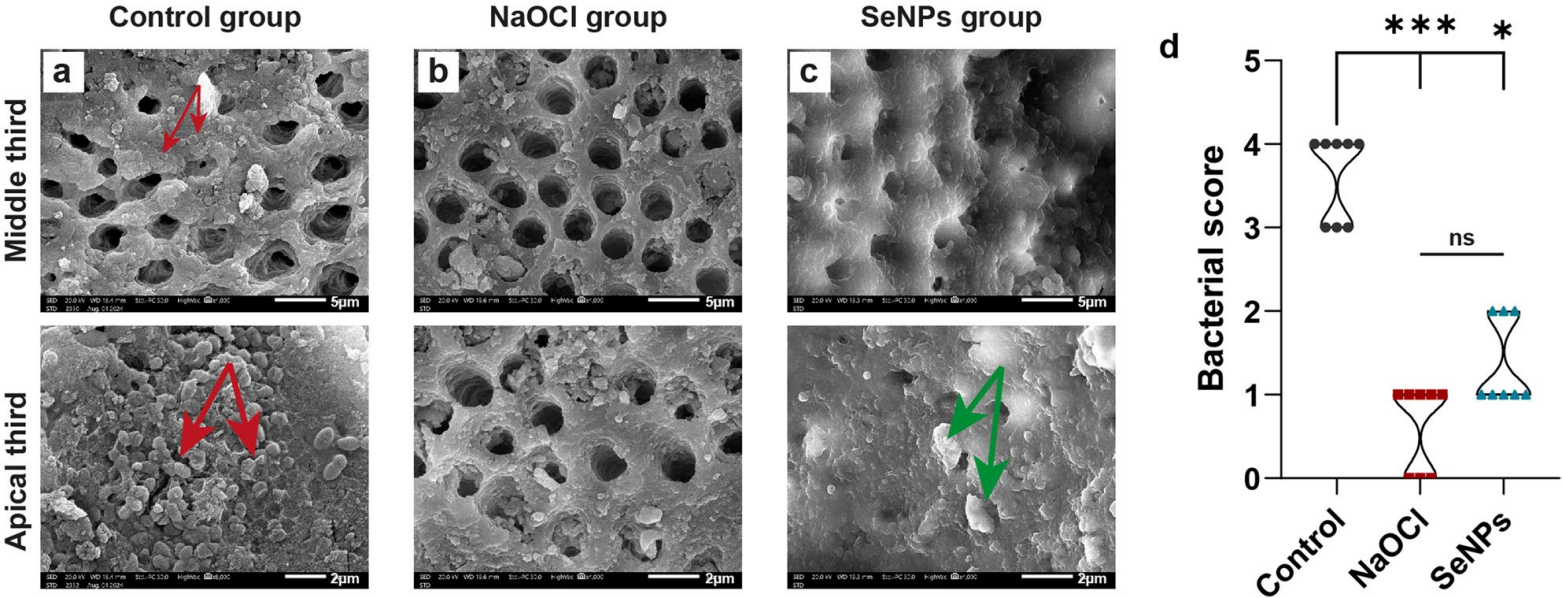
Assessment of dentinal surface morphology and bacterial presence following root canal irrigation. **a**-**c** Representative SEM micrographs (Scale bar = 5 μm for ×4000). **a **The control unirrigated group demonstrates bacterial colonization and exposure of the dentinal tubules. The presence of rod-shaped E. faecalis bacteria adherent to the dentinal surface and within tubules is evident (red arrows). **b **The NaOCl group reveals numerous open and clean dentinal tubules free of smear layer and debris in the middle and apical regions, indicating effective smear layer removal and deproteinization. **c **The SeNPs group at the middle and apical regions, respectively, exhibits a partially covered surface with granular aggregates of SeNPs (green arrows). The partially occluded tubules show clean surfaces with no observed bacterial structures. **d **A violin plot for the bacterial score (*n* = 8/group) analyzed by Kruskal-Wallis, followed by Dunn–Bonferroni post hoc analysis, reveals a significant reduction in the median score in the NaOCl*** (*p* < 0.001) and SeNPs* (*p* < 0.05) groups compared separately with the control unirrigated group. Meanwhile, ns (*p* > 0.05) marks the equivalent capacity in bacterial reduction between NaOCl and SeNPs

We performed a SEM analysis to assess bacterial colonization and dentinal surface morphology across experimental groups. The SEM images from the control group confirmed successful *E. faecalis* colonization following the 21-day incubation period. As shown in Fig. 3a, dense bacterial biofilm was observed covering the dentinal surface and partially occluding dentinal tubules. The bacteria exhibited coccoid morphology consistent with *E. faecalis*, forming organized microcolonies across the surface. The median biofilm score in this group was 4, indicating extensive biofilm formation and validating the bacterial inoculation protocol.

### Robust potentiality of SeNP irrigant combating E. faecalis

At 4000× magnification, SEM analysis of the NaOCl-treated group revealed an almost clean dentin surface with numerous open and patent dentinal tubules. NaOCl effectively removed the smear layer, exposing clean tubule walls and orifices. There was no observed surface debris or bacterial structures, indicating the high deproteinizing efficacy of NaOCl (Fig. 3b).

The SEM micrograph of the dentin treated with SeNPs displayed a partially covered dentinal surface with occluded tubules and scattered granular deposits. These nanostructures exhibited morphology consistent with SeNP clusters, adhering to the dentin matrix. Moreover, there were no morphologically distinguishable bacterial cells, suggesting the effective antibacterial action of SeNPs and a potential sealing effect over the dentinal tubules. The structural consistency with SeNPs and lack of visible bacterial colonies or coccal/rod forms reinforce the antimicrobial and tubule-blocking potential of selenium nanoparticles (Fig. 3c).

Overall, the SEM findings confirm that NaOCl is effective in the smear layer debridement while preserving the patency of dentinal tubules. Meanwhile, SeNPs provide a bioactive surface coating that can occlude tubules and inhibit microbial retention, supporting their potential dual role in antimicrobial protection and dentin sealing.

On a 5-point scale scoring of SEM-based bacterial presence, the unirrigated group recorded the highest bacterial scores (median = 4), confirming successful biofilm development. Following irrigation, the NaOCl group showed a significant reduction in bacterial presence (median = 1). Similarly, the SeNP group also exhibited a substantial decrease (median = 1). Statistical analysis using the Kruskal–Wallis test revealed a significant difference among the groups (H = 17.22, *p* < 0.001). These findings indicate that both irrigants were effective in reducing *E. faecalis* biofilm, with NaOCl exhibiting superior antimicrobial performance; however, SeNPs showed considerable antibacterial effects (Fig. 3d). Regarding the inter-examiner reliability, Cohen kappa coefficient demonstrated a substantial level of agreement between the two examiners (*κ* = 0.61, *p* < 0.001), indicating that the scoring consistency was significantly greater than chance.

## Discussion

The persistent nature of *E. faecalis* biofilms in post root canal treatment presents a significant clinical challenge, necessitating the development of novel irrigants with balanced antimicrobial efficacy and biocompatibility. NaOCl remains the gold standard irrigant owing to its potent antibacterial and tissue-dissolving properties. However, its cytotoxic effects, potential to induce dentinal erosion, and risk of periapical tissue damage have driven research toward safer, multifunctional alternatives [3, 42]. In line with these concerns, our findings revealed the drastic cytotoxic effect of NaOCl on oral epithelial cells and gingival fibroblasts, with remarkably low IC50 values of 0.004% and 0.007%, respectively. These results indicate that the commonly used 2.5% NaOCl is approximately 625-fold more potent than the tolerance threshold of oral cell lines. To address this limitation, this study investigated the efficacy of ascorbic acid-reduced PVP-stabilized SeNPs as a promising endodontic irrigant, leveraging the safety cellular profile combined with their favorable physicochemical characteristics, antimicrobial efficacy, and dentinal interactions.

The SeNPs in this study were chemically synthesized, using ascorbic acid (vitamin C) as a reducing agent. While biogenic synthesis offers biocompatible SeNPs, the biological reagents, including bacteria, fungi, and algae, may hinder the use of eco-friendly synthesized SeNPs in root canal irrigation. Additionally, the biosynthesis of SeNPs may be more expensive, as bacterial culture and isolation require specialized techniques, expertise, and specific equipment [43]. Therefore, chemical synthesis was accredited for SeNPs synthesis in the current study due to its efficiency and control over particle properties. For limiting the inherited toxicity by reducing agents, the adoption of ascorbic acid-mediated reduction not only offers an eco-friendly alternative but also leverages the antioxidant properties of SeNPs, potentially enhancing nanoparticle stability and biological performance [44].

Comprehensive characterization confirmed the favorable physicochemical profile of the synthesized SeNPs. A distinct blue shift in the UV–Vis absorption spectrum to 262 nm indicated successful nanoparticle formation, consistent with previous reports for ascorbic acid–reduced SeNPs [45]. Visualizing SeNPs by TEM revealed spherical nanoparticles with an average dry size of 93.15 nm, while DLS analysis confirmed a narrow size distribution. The difference in sizes between TEM and DLS reflects the technical variation in measuring dry and hydrodynamic nano sizes, respectively, where DLS increases the size by 20% [46, 47].

Biologically, the hydrodynamic size of nanoparticles, simulating their circulation in the body, is a pivotal factor in determining their activity and toxicity. Notably, the increases in hydrodynamic size should not be due to nanoparticle aggregation. Previously, in testing the antibacterial efficacy of silver nanoparticles, the aggregation of small-sized nanoparticles has led to the complete loss of their biological activity [48]. Our low PDI results, which were below the cutoff of 0.3 for aggregation [49], were evident in rejecting the aggregation of SeNPs, revealing the widely distributed nanoparticles. Although the zeta potential exhibited relatively low negative values, the PVP maintained nanoparticle stability, which promoted re-dispersibility after several weeks of storage in aqueous suspension at 4 °C and prevented aggregation, as an essential requirement for clinical application as an irrigant.

To further support the antimicrobial profile of SeNPs, the agar well diffusion assay demonstrated a clear inhibition zone of 18.3 ± 1.5 mm against *E. faecalis*. This diffusion-based approach confirmed the direct antibacterial action of SeNPs on solid media, consistent with the MIC findings. The formation of a defined inhibition zone around the SeNP-loaded well underscores the capacity of the nanoparticles to diffuse and exert localized antimicrobial activity, reinforcing their potential utility as a root canal irrigant [35].

The biological evaluation of SeNPs revealed a dual advantage in terms of antimicrobial efficacy and cytocompatibility. The MIC of 128 µg/mL against *E. faecalis* highlights the potent antibacterial activity of SeNPs, in agreement with earlier studies demonstrating their broad-spectrum antimicrobial potential [50, 51]. Importantly, cytotoxicity testing revealed a dose-dependent pattern, with an IC50 of 626 and 809 µg/mL against OECs and HGFs, respectively. A similar pattern has been reported in the cytocompatibility assessment of other antibacterial nanoplatforms, where primary HGFs displayed greater resistance than the more sensitive OECs when exposed to silver nanoparticles [52]. Notably, the observed variation in cellular susceptibility to SeNPs translated into a favorable antimicrobial selectivity index, ranging from 4.7- to 6.3-fold above the MIC, highlighting a substantial therapeutic window. Moreover, the recorded IC50s exceed cytotoxic concentrations previously reported for SeNPs against various cancer cell lines (23.20–393 µg/mL) [50, 53, 54], underscoring the selective safety profile of the synthesized nanoplatform toward healthy oral cells.

The antibacterial activity depends on the nature and structural composition of the bacterial cell wall and the interactions with the cell’s internal components. Multiple synergistic mechanisms underpin the antimicrobial efficacy of SeNPs. A primary mode of action involves the generation of ROS, which induces oxidative damage to bacterial membranes, proteins, and DNA, ultimately compromising cell viability [55, 56]. Additionally, SeNPs exhibit direct interactions with the bacterial cell envelope, disrupting membrane integrity, altering fluidity, and impairing essential transport processes [57]. Of particular relevance to endodontic applications, SeNPs possess the ability to penetrate biofilm matrices, which is an advantage over conventional irrigants that often fail to eliminate deeply embedded biofilm-associated bacteria [58]. Furthermore, selenium has been shown to interfere with bacterial metabolic pathways, including energy production and DNA replication, thereby exerting a multifaceted antimicrobial effect [59]. The nanoscale dimensions of SeNPs, combined with PVP-mediated stabilization, likely enhance their interaction with biofilms and microbial cells, contributing to the observed antibacterial outcomes.

While NaOCl remains the gold standard for root canal irrigation due to its potent antimicrobial and tissue-dissolving capabilities, its clinical use is associated with well-documented drawbacks. These include a potential for dentinal erosion and significant cytotoxicity to periapical tissues, as reflected by its drastic cytotoxic effect on the present OEC and HGFs, with severe consequences in cases of inadvertent extrusion beyond the apex [60]. Moreover, prolonged or repeated exposure to full-strength NaOCl has resulted in a significant reduction in dentin microhardness and an increase in susceptibility to collagen degradation, thereby predisposing teeth to structural weakening [61]. Reports have even linked NaOCl-induced dentin alterations with an increased risk of vertical root fractures, particularly under functional stresses [62].

In contrast, SeNPs offer a promising alternative by providing a relative antibacterial efficacy through the previously mentioned mechanisms while demonstrating a markedly wider safety margin, as evidenced by the high IC50 values against OEC and HGFs in the current study. Furthermore, unlike NaOCl, SeNPs possess inherent antioxidant and anti-inflammatory properties, which may contribute to minimizing collateral tissue damage and promoting periapical healing [56]. Their nanoscale dimensions and favorable surface characteristics facilitate deeper biofilm penetration, addressing a key limitation of conventional irrigants. Taken together, these attributes support the potential of SeNPs as a biocompatible and effective alternative to NaOCl in endodontic disinfection protocols [57].

SEM provided further validation of these experimental findings by offering a detailed visualization of the dentinal surface and assessing biofilm removal following irrigation. In the control group, SEM images confirmed extensive *E. faecalis* biofilm formation within the root canal system after a 21-day incubation period, verifying the success of the infection model. Post-irrigation SEM evaluation revealed observed bacterial reduction and dentinal surface alterations in both the SeNP and NaOCl groups.

Specifically, the NaOCl-treated specimens exhibited complete expulsion of the smear layer from dentinal surfaces with numerous open, patent tubules, consistent with NaOCl’s well-documented deproteinizing and smear layer removal capabilities [13]. However, such aggressive deproteinization raises concerns regarding the integrity of dentin microstructural and increased susceptibility to collagen degradation and microcracking [63]. In contrast, the SeNP-treated group pictured a distinct morphological pattern characterized by partial dentinal tubule occlusion and the presence of scattered granular deposits consistent with SeNP clusters. No morphologically distinguishable bacterial cells were observed in either group, suggesting comparable antibacterial efficacy.

Interestingly, the SeNP deposition on the dentinal surface may provide an additional clinical advantage by forming a bioactive layer that occludes dentinal tubules. This sealing effect could reduce dentinal permeability and inhibit bacterial re-infiltration, complementing the immediate antimicrobial action of SeNPs. Similar nanoparticle-based occlusion effects have been reported in other studies, reinforcing the plausibility of this dual-functionality [64].

In conclusion, the present study demonstrated that PVP-stabilized SeNPs, synthesized *via* ascorbic acid-mediated approach, exhibit potent antimicrobial activity against *E. faecalis*, a key pathogen implicated in endodontic treatment failures. Supported by polymeric surface coating, the synthesized SeNPs exhibited favorable physicochemical characteristics, including size, stability, and uniform morphology. The biological evaluation revealed an antibacterial selectivity potential of SeNPs with a favorable safety margin, as reflected by the higher IC50 values for oral epithelial and gingival cells compared to the concentration required to inhibit *E. faecalis*, addressing key concerns associated with conventional irrigants. Moreover, boosting the antibacterial selectivity index of SeNPs can be achieved through functionalization with antimicrobial peptides that preferentially target the more negatively charged bacterial membranes while sparing mammalian cells, depending on the cationic properties of the peptides [65].

SEM analysis further demonstrated the relative antibacterial activity of SeNPs in disrupting biofilms and decontaminating dentinal surfaces, although their antibacterial efficacy did not fully match that of the gold-standard NaOCl. Nonetheless, the substantial biocompatibility of SeNPs underscores their potential as a safe sealant for dentinal tubules. To substantiate this proposed mechanism, topographic characterization of SeNPs-treated dentine surfaces using one of the elemental mapping techniques would be valuable in confirming the deposition and retention of SeNPs within dentinal tubules. In this context, Energy-Dispersive X-ray (EDX) analysis is one of the complementary techniques that integrates with SEM in topographic analysis of sealant penetration, together with providing the elemental composition of the dentin surface and sealant [66, 67]. Moreover, the use of confocal laser scanning microscopy (CLSM) coupled with live/dead staining would significantly strengthen the evaluation by enabling precise, automated quantification of bacterial viability through advanced image analysis.

Among the technical limitations in this study is the use of straight canals, while ensuring reproducibility, does not reflect the anatomical complexity of curved and multi-rooted molars. Therefore, future studies should evaluate SeNPs in molar models for greater clinical relevance. Second, the pre-inoculation use of EDTA facilitated tubule penetration but may alter nanoparticle stability and antimicrobial performance if applied concurrently, requiring further investigation. Finally, standardized irrigant neutralization, such as sodium thiosulfate for NaOCl, was not employed to preserve SeNP–dentin interactions for SEM analysis, which may have influenced bacterial recovery. Incorporating validated neutralization and culture-based methods in future work will help optimize irrigation protocols and strengthen microbiological validity.

While further quantitative, in vivo, and long-term biocompatibility studies are warranted to establish clinical applicability, these findings position SeNPs as a promising multifunctional nanoplatform for improving endodontic disinfection protocols. Future research exploring their impact on dentinal microstructure, obturation compatibility, and biofilm reformation is essential to advance their translation into clinical practice. Moreover, evaluating the long-term antibacterial efficacy and release profile of SeNPs will be critical for their potential incorporation into obturating gutta-percha, thereby providing not only enhanced mechanical resilience but also sustained antibacterial protection.

## Data Availability

The datasets used and/or analyzed during the current study are available from the corresponding author on reasonable request.
